# Systematic Review and Cumulative Analysis of the Combination of Mitomycin C plus Bacillus Calmette-Guérin (BCG) for Non–Muscle-Invasive Bladder Cancer

**DOI:** 10.1038/s41598-017-03421-5

**Published:** 2017-06-09

**Authors:** Tuo Deng, Bing Liu, Xiaolu Duan, Tao Zhang, Chao Cai, Guohua Zeng

**Affiliations:** 1 Department of Urology, Minimally Invasive Surgery Center, The First Affiliated Hospital of Guangzhou Medical University, Guangzhou, China; 2 Guangzhou Institute of Urology, Guangzhou, China; 3 Guangdong Key Laboratory of Urology, Guangzhou, China; 4 The First Affiliated Hospital of Jinan University, Guangzhou, China

## Abstract

This systematic review and cumulative analysis aimed to explore the efficacy and safety of the combination of intravesical mitomycin C (MMC) plus bacillus Calmette-Guerin (BCG) for non-muscle-invasive bladder cancer (NMIBC) patients. A comprehensive literature search using Pubmed, Embase, Medline, Cochrane Library, CBM, CNKI and VIP databases was performed to identify studies applying intravesical MMC plus BCG therapy on NMIBC patients up to June 2016. Summarized unadjusted odds ratios (ORs) with 95% confidence intervals (CIs) were calculated to assess the efficacy and safety of the combination therapy. A total of 25 studies containing 2749 NMIBC patients were included in this systematic review. Compared with BCG monotherapy, the combination therapy could significantly reduce the tumor recurrence rate (OR = 0.64, 95% CI: 0.44–0.94, *P* = 0.02) and cancer-specific mortality (OR = 0.54, 95% CI: 0.34–0.87, *P* = 0.01), without more toxicities (OR = 0.58, 95% CI: 0.17–1.94, *P* = 0.37). The combination therapy could also lead to significant lower tumor recurrence rate than MMC monotherapy (OR = 0.41, 95% CI: 0.24–0.69, *P* = 0.0009). Our study indicates that the combination of MMC plus BCG instillation is an effective and safe adjuvant treatment for NMIBC patients.

## Introduction

Urinary bladder cancer is one of the most common malignant tumors all over the world, occupying about 4% of the cancer. The incidence of bladder cancer was approximately 7%^1^, ranked fourth among male tumors. 70% of these patients suffer from superficial or non-muscle-invasive tumors^2^. Transurethral resection (TUR) is the current primary treatment. Subsequent intravesical adjuvant treatments including chemotherapy and immunotherapy are recommended to reduce the recurrence rate and delay the progression of the tumor^3, 4^.

Most widely used adjuvant agents are bacillus Calmette-Guerin (BCG) and mitomycin C (MMC), especially for tumors with intermediate to high recurrence or progression rate^5^. Intravesical BCG is the most recommended treatment for non-muscle-invasive bladder cancer (NMIBC) with a relative satisfactory effect according to EAU guidelines^6^. However, the recurrent rate is still up to 60–70% and 30% of tumors turn out to be higher grade^7^. Therefore, advanced adjuvant regimens are necessary to improve the efficacy. Combination of intravesical MMC plus BCG instillation, a novel adjuvant therapy, has been researched in a variety of studies and showed a more enhanced antitumor effect^8, 9^. Detailed combined regimens, drug doses and therapeutic courses varied among these studies, which brought different results^10–13^.

No guidelines or protocols have been made yet to recommend the combination of intravesical MMC and BCG therapy for NMIBC. And as far as we have concerned, few conclusive articles or reviews focused on the efficacy and safety of various intravesical MMC plus BCG therapies on NMIBC patients. Consequently, we conducted this systematic review and cumulative analysis based on all relevant original studies and aimed to provide further instructions for adjuvant treatments of NMIBC.

## Results

### Eligible studies and characteristics

**Figure 1:**
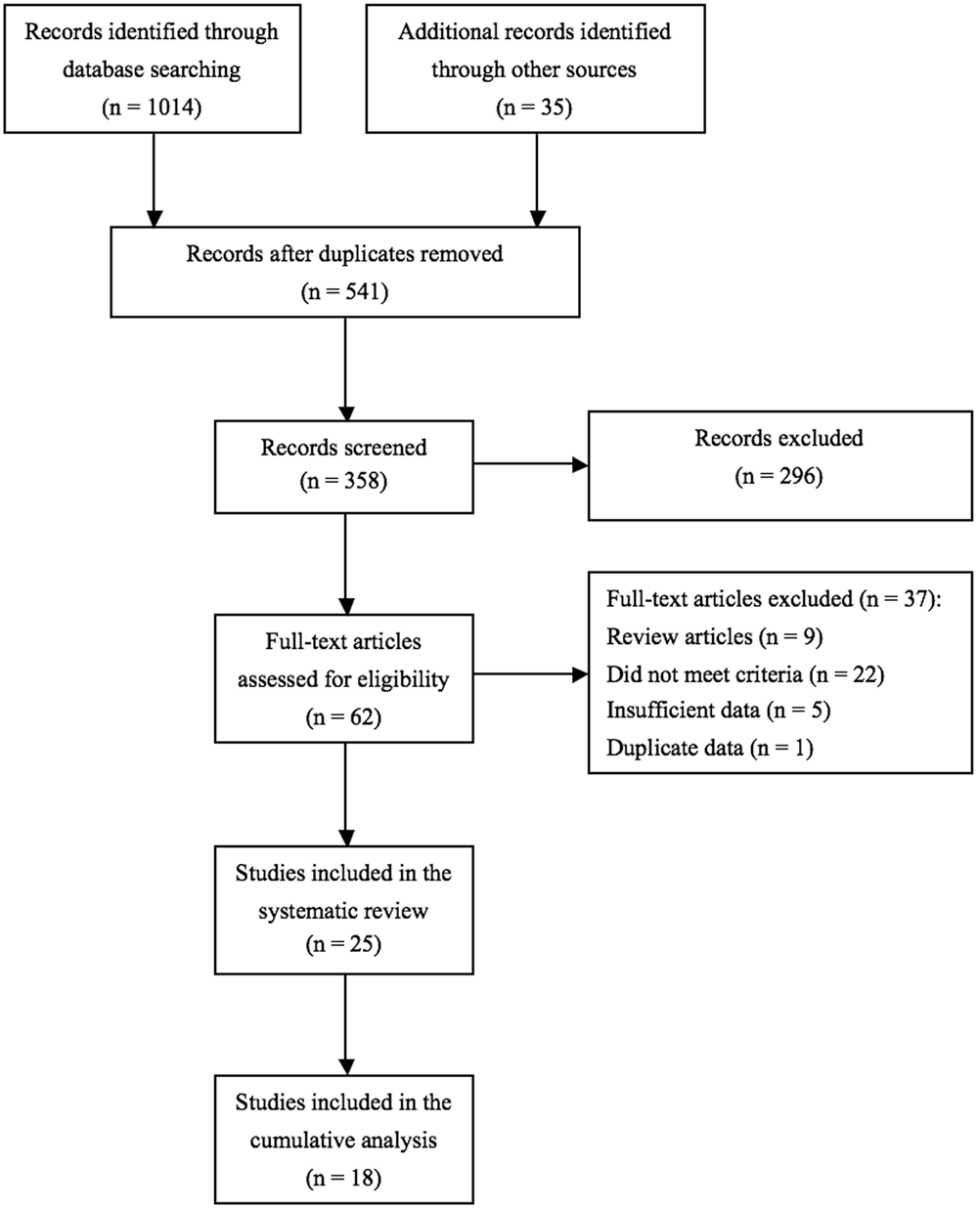
Flow diagram of the systematic review.

25 studies^10–34^ containing 2749 NMIBC patients were included in this systematic review (Fig. 1). Baseline characteristics of all eligible studies were shown in Table 1. Among 25 included studies, 16 were randomized controlled trials (RCTs)^10–13, 15–21, 24, 28, 31, 33, 34^, 4 were retrospective comparative trials^14, 26, 27, 29^, 1 was retrospective cohort study^30^ and remaining 4 were clinical series^22, 23, 32, 25^.

**Table 1:** The baseline characteristics of included studies.

Combination regimen of MMC+BCG	Reference	Country	Ethnicity	Recruitment period	Study design	LOE	Tumor stage	No. of cases receiving MMC+BCG	Mean/median age(yr)	Mean/median follow-up time (mo)
A single dose of perioperative MMC prior to instillation with BCG										
	Badalato *et al*.^14^	USA	mixed	2000–2010	Retrospective comparative trial	3	Ta, T1, Tis	48	69.6	33
	Gülpinar *et al*.^10^	Turkey	Europeans	2004–2006	RCT	2b	Ta, T1	25	58.2	41.3
	Ye *et al*.^15^	China	Asian	1997–2002	RCT	2b	Ta, T1	50	57	32
	Weiss *et al*.^29^	USA	mixed	1977–2009	Retrospective comparative trial	3	Ta, T1, Tis	23	—	54
Sequential instillation with MMC and BCG										
	Di Stasi *et al*.^11^	Italy	Europeans	1994–2002	RCT	2b	T1, Tis	107	66	91
	Oosterlinck *et al*.^16^	Multi-country in Europe	Europeans	2001–2005	RCT	1b	Ta, T1, Tis	41	68	56.4
	He *et al*.^17^	China	Asians	2005–2009	RCT	2b	Ta, T1	40	61.2	21.2
	Liu *et al*.^18^	China	Asians	2000–2003	RCT	2b	Ta, T1	59	55	35
	Ma *et al*.^19^	China	Asians	1996–1998	RCT	2b	—	29	52	37.9
	Kaasinen *et al*.^20^ and Järvinen *et al*.^21^	Finland	Europeans	1992–1996	RCT	2a	Ta, T1	102	68	30.7
										117.6
	Svatek *et al*.^22^	USA	mixed	—	Case series	4	Ta, T1, Tis	12	67	21.4
	Cai *et al*.^23^	China	Asians	2007–2011	Case series	4	Ta, T1	30	60.3	20.4
	Gan *et al*.^30^	UK	Europeans	2009–2013	Retrospective cohort study	3	Ta, T1, Tis	104	68	24
	Witjes *et al*.^31^	Netherlands	Europeans	1991–1993	RCT	2a	Ta, T1, Tis	90	—	32
	Van der Meijden *et al*.^32^	Netherlands	Europeans	1990–1992	Case series	4	Ta, T1	35	70	19.8
Alternating instillation with MMC and BCG										
	Rintala *et al*.^33^ and Järvinen *et al*.^12^	Finland	Europeans	1987–1992	RCT	2a	Ta, T1, Tis	28	66	33
										86.4
	Kaasinen *et al*.^24^	Finland, Norway and Sweden	Europeans	1992–1997	RCT	2a	Ta, T1, Tis	159	71	56.3
	Zhang *et al*.^25^ and Sun *et al*.^26^	China	Asians	1998–2006	Retrospective comparative trial	3	Ta, T1	32	62.5	28
	Bao *et al*.^27^	China	Asians	1999–2006	Retrospective comparative trial	3	Ta, T1, Tis	20	70	24
	Rintala *et al*.^34^	Finland	Europeans	1987–1992	RCT	2a	Ta, T1	95	68.5	34
Mixed instillation with MMC plus BCG										
	Solsona *et al*.^13^	Spain	Europeans	1993–1994	RCT	1b	Ta, T1, Tis	211	65	85.2
	Fang *et al*.^28^	China	Asians	1999–2000	RCT	2a	Ta, T1	21	67.5	23.4

BCG = bacillus Calmette-Guerin; LOE = level of evidence; MMC = mitomycin C; RCT = randomized controlled trial.

In all studies, 18^10–19, 24, 26–29, 31, 33, 34^ were included in our cumulative analysis, comparing the efficacy of combined MMC plus BCG therapy with MMC or BCG monotherapy on NMIBC patients. Among them, MMC + BCG versus BCG alone was conducted in 10 studies^10, 11, 13, 14, 16–18, 24, 27, 29^, MMC + BCG versus MMC alone was referred in 7 studies^12, 15, 19, 28, 31, 33, 34^ and the rest 1^26^ compared MMC + BCG with either MMC or BCG.

### Quality assessments of included studies

**Figure 2:**
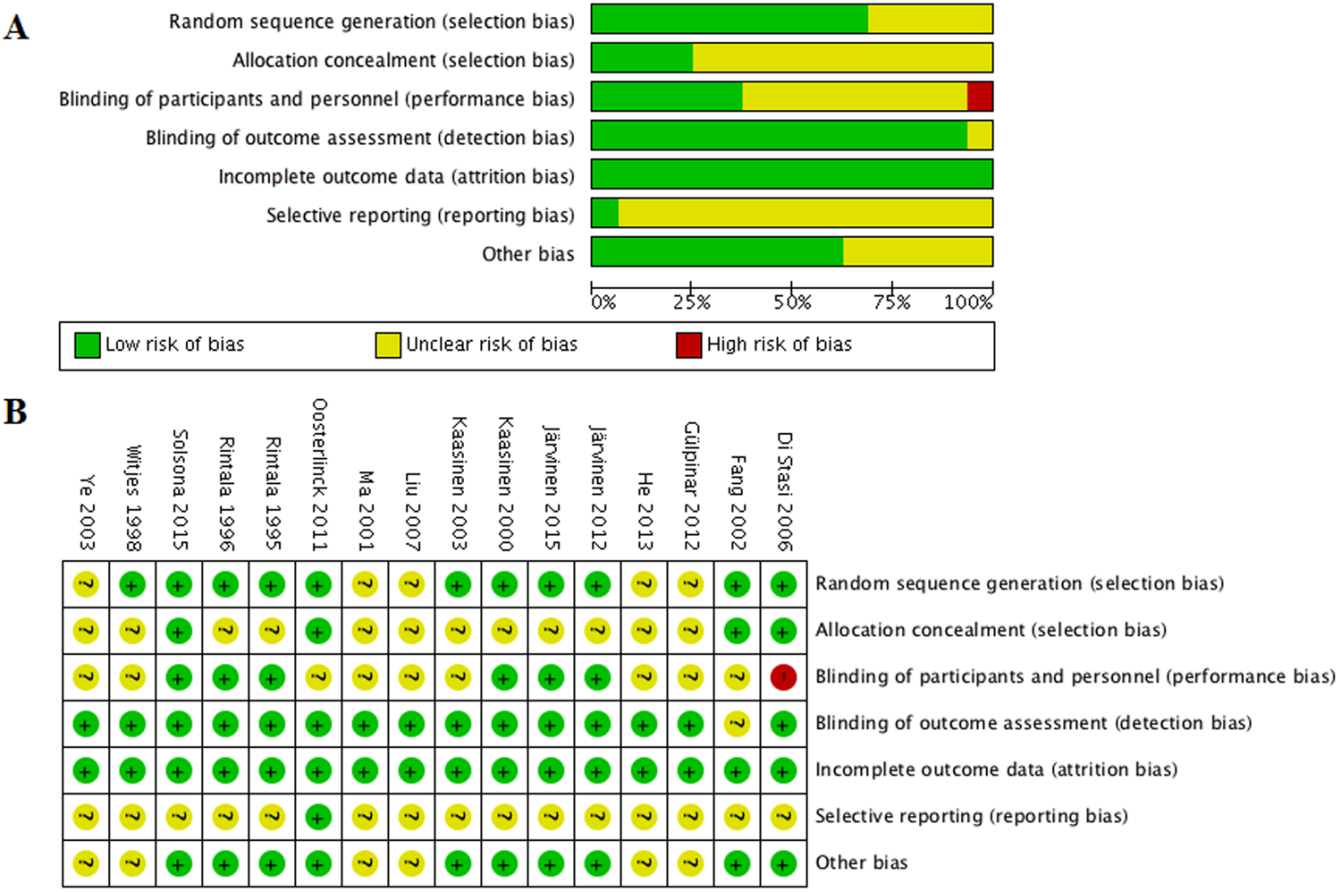
Assessment of bias risk for included RCTs. (**A**) Methodological quality graph: authors’ judgments about each methodological quality item presented as percentages across all included studies; (**B**) Methodological quality summary: authors’ judgments about each methodological quality item for each included study, “+” low risk of bias; “?” unclear risk of bias; “−” high risk of bias).

Level of evidence (LOE) was accessed for all 25 included studies and results were listed in Table 1. Among 16 RCTs, 9 were in low risk of bias^12, 13, 16, 20, 21, 24, 28, 33, 34^, 6 were in moderate risk of bias^10, 15, 17–19, 31^ and the remaining one was in high risk of bias^11^ according to the quality assessment (Fig. 2). However, the risk of detection and attrition biases were low in all of them. Additionally, 7 RCTs were in relative high quality^12, 13, 16, 20, 21, 33, 34^.

### Instillation regimens and prognoses of intravesical MMC plus BCG

1361 NMIBC patients from 25 eligible studies received intravesical MMC plus BCG instillation as an adjuvant therapy besides surgery (Table 2). Combination regimens in these studies could be divided into four subtypes: single dose of perioperative MMC prior to BCG (combination regimen 1) was applied in 4 studies^10, 14, 15, 29^; sequential instillations with MMC and BCG (combination regimen 2) were used in 12 studies^11, 16–23, 30–32^; 7 studies^12, 24–27, 33, 34^ adopted alternating instillations with MMC and BCG (combination regimen 3); and last 2 studies^13, 28^ preferred mixed instillations with MMC plus BCG (combination regimen 4). Table 3 showed prognoses of patients receiving combination therapies in all included studies according to different instillation regimen and follow-up time.

**Table 2:** Detailed outcomes of patients receiving combination therapy.

Combination regimen of MMC + BCG	Reference	No. of case receiving MMC + BCG	Mean/median age(yr)	Mean/median follow-up time (mo)	During follow-up time					5-year recurrence-free survival rate	No. severe side-effects (%)
					No. recurrence (%)	No. disease-free case (%)	No. progression (%)	No. death from any causes (%)	No. death from bladder cancer (%)		
A single dose of perioperative MMC prior to instillation with BCG											
	Badalato *et al*.^14^	48	69.6	33	21 (43.8)	27 (56.2)	—	—	—	56.3%	—
	Gülpinar *et al*.^10^	25	58.2	41.3	9 (36)	16 (64)	1 (4)	—	—	—	0
	Ye *et al*.^15^	50	57	32	3 (6)	—	0	—	0	—	0
	Weiss *et al*.^29^	23	—	54	10 (43.5)	10 (43.5)	—	3 (13)	—	48.4%	—
Sequential instillation with MMC and BCG											
	Di Stasi *et al*.^11^	107	66	91	45 (42.1)	62 (57.9)	10 (9.3)	23 (21.5)	6 (5.6)	—	3 (2.8)
	Oosterlinck *et al*.^16^	41	68	56.4	23 (56.1)	25 (61)	3 (7.3)	7 (17.1)	0	51.4%	5 (12.2)
	He *et al*.^17^	40	61.2	21.2	5 (12.5)	—	—	—	0	—	0
	Liu *et al*.^18^	59	55	35	9 (15.3)	—	3 (5.1)	—	0	—	0
	Ma *et al*.^19^	29	52	37.9	3 (10.3)	—	—	—	—	—	0
	Kaasinen *et al*.^20^ and Järvinen *et al*.^21^	102	68	30.7	14 (13.7)	73 (71.6)	3 (2.9)	4 (3.9)	0	67%	2 (2)
				117.6	44 (43.1)	32 (31.4)	6 (5.9)	56 (54.9)	3 (2.9)	—	
	Svatek *et al*.^22^	12	67	21.4	1 (8.3)	11 (91.7)	0	0	0	—	0
	Cai *et al*.^23^	30	60.3	20.4	4 (13.3)	26 (86.7)	0	0	0	—	0
	Gan *et al*.^30^	104	68	24	31 (29.8)	66 (63.5)	5 (4.8)	1 (1)	0	—	1 (1)
	Witjes *et al*.^31^	90	—	32	35 (38.9)	47 (52.2)	5 (5.6)	21 (23.3)	5 (5.6)	52.2%	16 (17.8)
	Van der Meijden *et al*.^32^	35	70	19.8	8 (22.9)	—	1 (2.9)	0	0	—	2 (5.7)
Alternating instillation with MMC and BCG											
	Rintala *et al*.^33^ and Järvinen *et al*.^12^	28	66	33	6 (21.4)	14 (50)	2 (7.1)	0	0	—	0
				86.4	19 (67.9)	—	8 (28.6)	20 (71.4)	8 (28.6)	34%	—
	Kaasinen *et al*.^24^	159	71	56.3	71 (44.7)	72 (45.3)	34 (21.4)	—	13 (8.2)	40.7%	10 (6.3)
	Zhang *et al*.^25^ and Sun *et al*.^26^	32	62.5	28	2 (6.3)	30 (93.7)	0	0	0	—	0
	Bao *et al*.^27^	20	70	24	0	—	0	—	0	—	0
	Rintala *et al*.^34^	95	68.5	34	57 (60)	38 (40)	3 (3.2)	2 (2.1)	0	—	6 (6.3)
Mixed instillation with MMC plus BCG											
	Solsona *et al*.^13^	211	65	85.2	44 (20.9)	—	26 (12.3)	51 (24.2)	10 (4.7)	—	20 (9.5)
	Fang *et al*.^28^	21	67.5	23.4	1 (4.8)	—	—	—	0	—	0

BCG = bacillus Calmette-Guerin; MMC = mitomycin C.

**Table 3:** Patients’ prognoses with different combination regimen and follow-up time.

Combination regimen	Follow-up time	No. of included study	No. recurrence/total (%)	No. disease-free case/total (%)	No. progression/total (%)	No. death from any causes/total (%)	No. death from bladder cancer/total (%)	No. severe side-effects/total (%)
Combination regimen 1	Medium-term (2–5 yrs)	4	43/146 (29.5)	53/96 (55.2)	1/75 (1.3)	3/23 (13)	0/50	0/75
Combination regimen 2	Short-term (≤2 yrs)	5	49/221 (22.2)	103/146 (70.5)	6/181 (3.3)	1/181 (0.6)	0/221	3/221 (1.4)
	Medium-term (2–5 yrs)	5	84/321 (26.2)	145/233 (62.2)	14/292 (4.8)	32/233 (13.7)	5/292 (1.7)	23/321 (7.2)
	Long-term (≥5 yrs)	2	89/209 (42.6)	94/209 (45)	16/209 (7.7)	79/209 (37.8)	9/209 (4.3)	5/209 (2.4)
Combination regimen 3	Short-term (≤2 yrs)	1	0/20	—	0/20	—	0/20	0/20
	Medium-term (2–5 yrs)	5	136/314 (43.3)	154/314 (49)	39/314 (12.4)	2/155 (1.3)	13/314 (4.1)	16/314 (5.1)
	Long-term (≥5 yrs)	1	19/28 (67.9)	—	8/28 (28.6)	20/28 (71.4)	8/28 (28.6)	—
Combination regimen 4	Short-term (≤2 yrs)	1	1/21 (4.8)	—	—	—	0/21	0/21
	Long-term (≥5 yrs)	1	44/211 (20.9)	—	26/211 (12.3)	51/211 (24.2)	10/211 (4.7)	20/211 (9.5)

Combination regimen 1: a single dose of perioperative MMC prior to BCG; Combination regimen 2: sequential instillation with MMC and BCG; Combination regimen 3: alternating instillation with MMC and BCG; Combination regimen 4: mixed instillation with MMC plus BCG.

### MMC plus BCG instillation versus BCG alone

11 studies compared the efficacy of MMC plus BCG with BCG alone instillation (Supplementary Table 1).

### Recurrence

**Figure 3:**
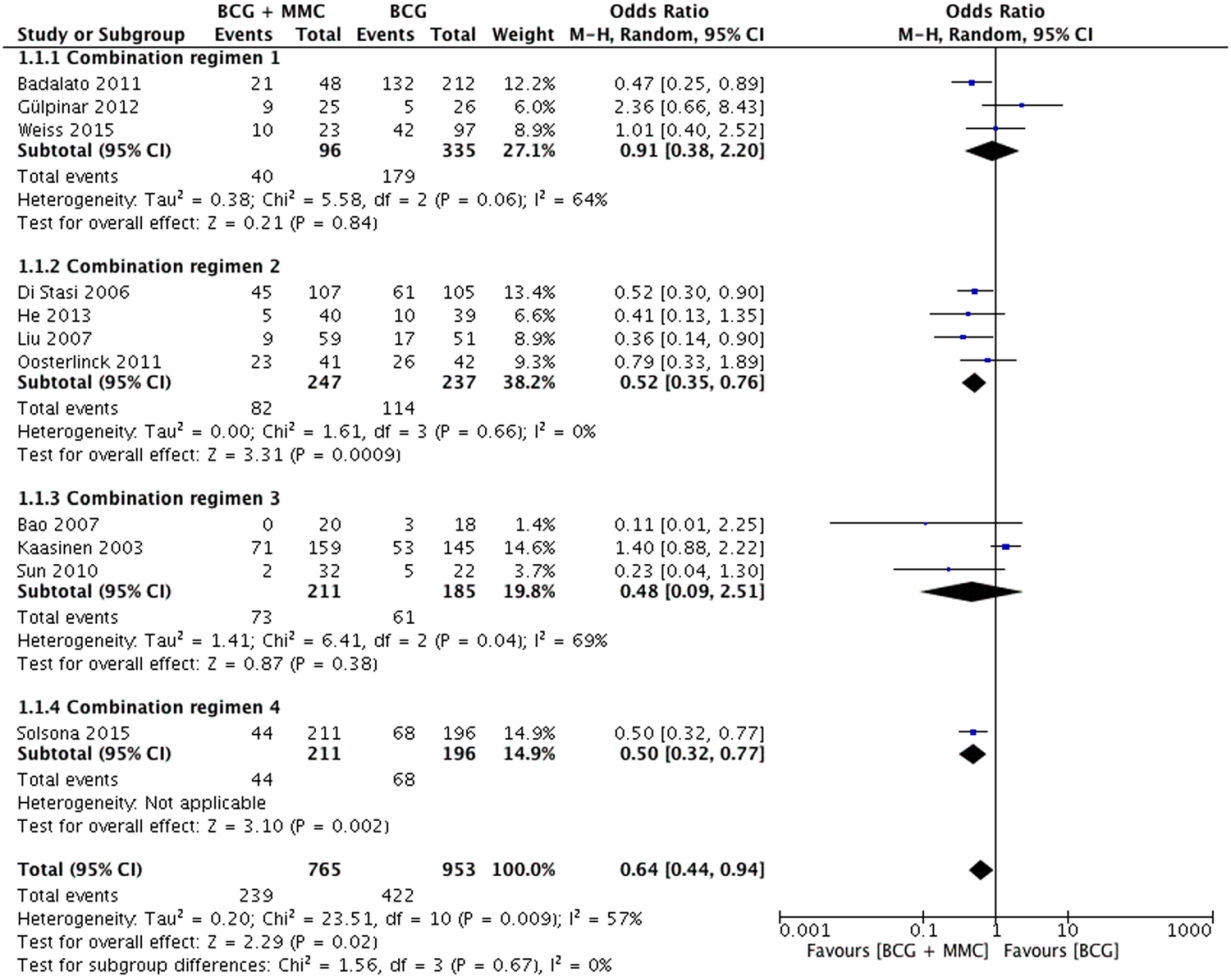
Forest plot of tumor recurrence rate comparing combination therapy with BCG monotherapy.

Tumor recurrence rate was compared between intravesical MMC plus BCG instillation and BCG alone treatment among NMIBC patients in all 11 studies. Slight heterogeneity was observed (*I* ^2^ = 57%, *P* = 0.009), and the recurrence rate in patients receiving MMC + BCG was significantly lower than BCG alone [odds ratio (OR) = 0.64, 95% confidence interval (CI): 0.44–0.94, *P* = 0.02) (Fig. 3). In subgroup analyses, patients in following subgroups also benefited more from MMC + BCG instillations significantly than BCG alone: retrospective comparative trials, combination regimen 2, combination regimen 4, short-term and long-term follow-ups, Asians populations, therapeutic courses ≤1yr and >2 yrs, and instillation numbers ≥24 (Supplementary Table 2). No publication bias was detected through both inverted funnel plot and Egger’s test (t = −1.65, *P* = 0.138).

In 3 studies, we reported multivariable adjusted hazard ratios (HRs) to prevent tumor recurrence of combined MMC and BCG instillation compared with BCG alone. No significant difference was found between two groups (HR = 0.86, 95% CI: 0.50–1.49, *P* = 0.59) with moderate heterogeneity (*I* ^2^ = 80%, *P* = 0.007).

### Disease-free survival

Number of disease-free patients during follow-up time was mentioned in 6 studies, and slight heterogeneity was found (*I* ^2^ = 67%, *P* = 0.01). Although no significant difference was observed between MMC + BCG and BCG groups (OR = 1.16, 95% CI: 0.70–1.92, *P* = 0.56), patients receiving combination regimen 2 shared a significant higher disease-free survival rate than BCG alone (OR = 1.76, 95% CI: 1.11–2.79, *P* = 0.02) without heterogeneity (*I* ^2^ = 0%, *P* = 0.57) (Supplementary Fig. 1). No publication bias was detected through the inverted funnel plot.

### Progression

**Figure 4:**
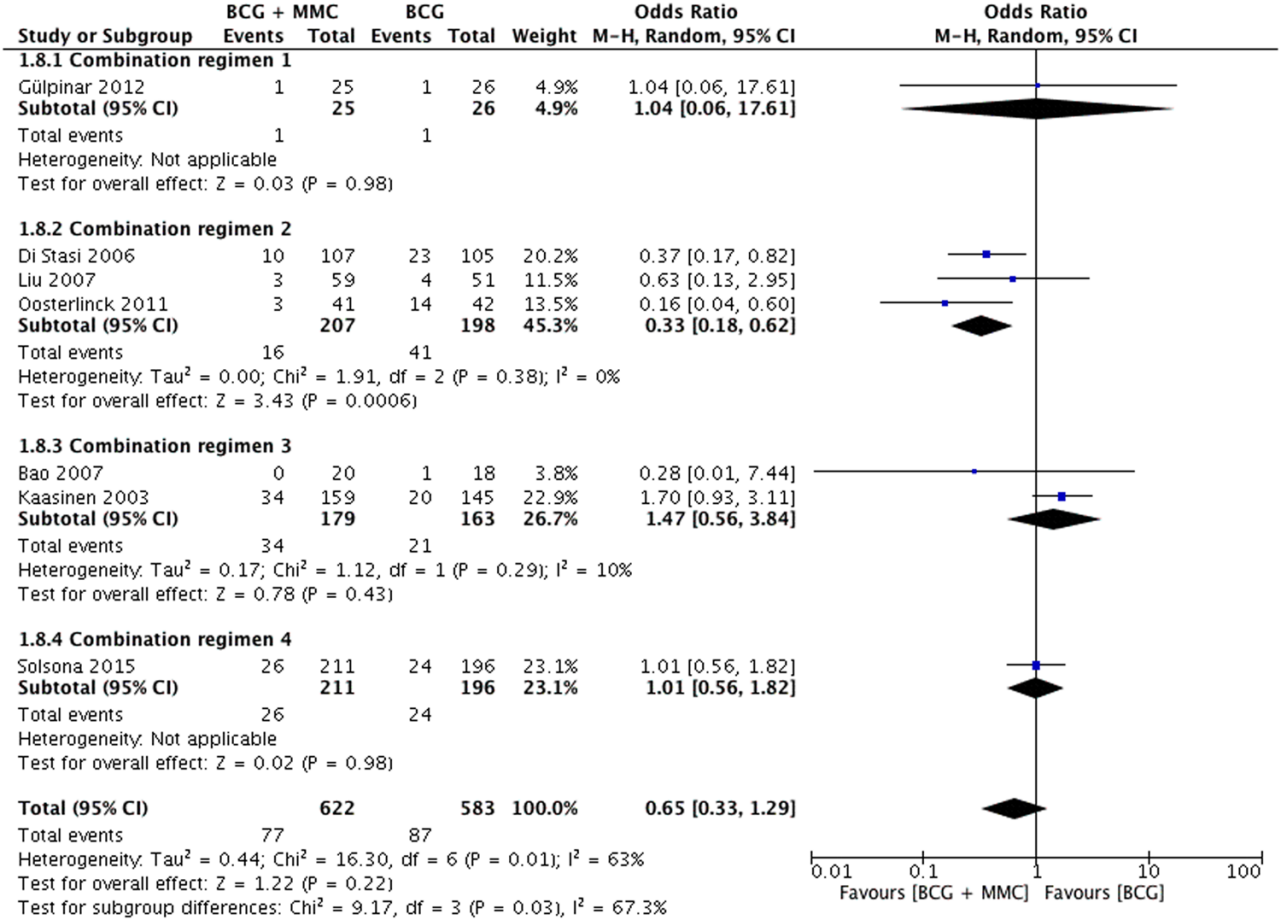
Forest plot of tumor progression rate comparing combination therapy with BCG monotherapy.

7 studies compared rate of tumor progression between MMC + BCG and BCG alone groups. No significant difference occurred (OR = 0.65, 95% CI: 0.33–1.29, *P* = 0.22) with slight heterogeneity (*I* ^2^ = 63%, *P* = 0.01). However, subgroup analyses indicated that the application of combination regimen 2 could significantly reduce the risk of progression for NMIBC patients compared with BCG alone (OR = 0.32, 95% CI: 0.18–0.60, *P* = 0.0004) bearing no heterogeneity among these relevant studies (*I* ^2^ = 0%, *P* = 0.38) (Fig. 4). The inverted funnel plot did not demonstrate any indication of publication bias.

### Cancer-specific mortality

During the follow-up time, patients who died from bladder cancer were reported in 5 studies. Significant lower cancer-specific mortality was discovered in MMC + BCG compared with BCG group (OR = 0.54, 95% CI: 0.34–0.87, *P* = 0.01) sharing no heterogeneity (*I* ^2^ = 41%, *P* = 0.15) (Supplementary Fig. 2). Furthermore, significant advantage was only tested in combination regimen 2 (OR = 0.24, 95% CI: 0.10–0.59, *P* = 0.002) after subgroup analyses were conducted. The inverted funnel plot showed no publication bias.

### Severe side-effects

Toxicities of intravesical MMC + BCG versus BCG alone therapies were assessed in 5 studies with moderate heterogeneity (*I* ^2^ = 80%, *P* = 0.0004). Combination of intravesical MMC + BCG instillation did not seem to bring fewer toxicities than BCG alone (OR = 0.58, 95% CI: 0.17–1.94, *P* = 0.37) (Supplementary Fig. 3). Nevertheless, subgroup analyses indicated that combination regimen 3 could significantly decrease the toxicity of combination therapy compared with BCG monotherapy (OR = 0.18, 95% CI: 0.09–0.38, *P* < 0.00001). No publication bias was detected through the inverted funnel plot.

### MMC+BCG instillation versus MMC alone

**Figure 5:**
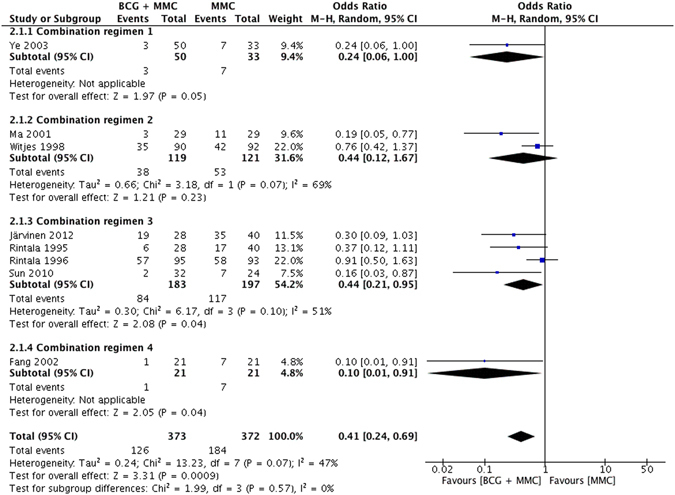
Forest plot of tumor recurrence rate comparing combination therapy with MMC monotherapy.

8 studies concentrated on the efficacy of intravesical MMC + BCG instillation versus MMC alone on NMIBC patients (Supplementary Table 3). Recurrence rate was compared in all 8 studies, and our results indicated that combined intravesical therapy was significantly more effective to decrease tumor recurrence than MMC alone (OR = 0.41, 95% CI: 0.24–0.69, *P* = 0.0009) with slight heterogeneity (*I* ^2^ = 47%, *P* = 0.07) (Fig. 5). In addition, 5 studies compared the tumor progression rate between MMC + BCG and MMC alone groups with no significant difference (OR = 0.83, 95% CI: 0.43–1.59, *P* = 0.57) (Supplementary Fig. 4). No heterogeneity existed either (*I* ^2^ = 0%, *P* = 0.88). Comparison of toxicities between two groups was also conducted in 5 studies and no significant difference was observed (OR = 1.18, 95% CI: 0.63–2.19, *P* = 0.61) (Supplementary Fig. 5). Cancer-specific mortality was only reported in 2 studies, and no significant difference was discovered.

## Discussion

This systematic review aimed to evaluate the efficacy and safety of combined intravesical MMC plus BCG instillation as a novel adjuvant therapy for NMIBC. Our analyses concluded that, compared with BCG or MMC monotherapy, the combination therapy could reduce the recurrence rate of NMIBC significantly without causing more toxicities. As a result, all evidences we have achieved till now support that combined intravesical MMC plus BCG instillation may be a better choice for NMIBC patients.

Previous studies have shown that the adherence to bladder wall of BCG is an important step for immunotherapy^35, 36^. Chemical disruption of the bladder urothelium induced by MMC could enable BCG to attach more efficiently to bladder wall and then improve the immune response and antitumor activity^37^. Furthermore, MMC instillation could also promote BCG uptake and activate related immune effector cells^38–40^. Therefore, an enhanced antitumor effect could be achieved by combined intravesical MMC and BCG instillation.

So far, several studies^8–13^ have investigated the antitumor effect of combined intravesical MMC plus BCG instillation. Lan *et al*.^41^ recently conducted a meta-analysis including only RCTs, having compared the efficacy of combined BCG and MMC therapy with each monotherapy on NMIBC patients. Results from 8 RCTs in their study showed a significant decreased recurrence rate in patients receiving combination therapy compared with monotherapy. However, since a lot of comparative and cohort studies were not included, their conclusions appeared to be rigorous to some extent. Some animal experiments also drew a similar conclusion with us^22, 42^. Matsushima *et al*.^42^ found MMC plus BCG treatment could inhibit tumor growth and cellular proliferation, and prolong the survival period compared to the BCG-alone therapy through an orthotopic bladder cancer model. Moreover, Svatek *et al*.^22^ identified macrophages were polarized toward a beneficial M1 phenotype after MMC plus BCG instillation in a murine model of bladder cancer, which indicated the antitumor effect of combination instillation could be improved by increased number of beneficial cells.

In this systematic review, we recognized that different combination regimens were carried out in these studies, which might have caused varied effects. Table 3 showed that combination regimen 4 could reduce recurrence but lead to more severe side-effects than others. While considering delaying tumor progression and reducing cancer-specific mortality in long-term follow-up, combination regimen 2 might be a better choice. Several courses of MMC before sequential BCG instillation could not only improve the antitumor function, but also promote the activation and production of immune effector cells^38–40^. Nevertheless, since these findings were not obtained by statistical comparisons and cumulative analyses of different combination regimens, they should only represent our own opinions and could not be regarded as evidential results.

Solsona *et al*.^13^ conducted a RCT demonstrating that combined MMC plus BCG therapy was more toxic than BCG alone with severe side-effects rate of 9.5%. However, our analysis indicated that taking all clinical trials into consideration, combination therapy did not cause more toxicities than BCG or MMC monotherapy. Therefore, combination of MMC plus BCG treatment seems to be safe, while more clinical studies are still needed for further evaluation.

Several potential limitations should be addressed about this analysis. First, included studies lasted a time span as long as 21 years, during which the living environment and quality of life might change. Second, data of some studies was incomplete even by contacting authors. Third, most high-quality trials were conducted in Europeans and Asians, which might restrict the application of our results on other populations. At last, insufficient numbers of related studies might bring some potential bias to our results.

## Conclusion

Our study concluded that combination of MMC plus BCG intravesical instillation was an effective and safe adjuvant treatment for NMIBC patients after TUR. This therapy could significantly reduce the tumor recurrence rate and would not bring more toxicities than BCG or MMC monotherapy. However, further high-quality clinical trials are still needed to verify conclusions of our study.

## Materials and Methods

### Search strategy

A systematic literature search using Pubmed, Embase, Medline, Cochrane Library, CBM, CNKI and VIP databases was performed to identify studies exploring the efficacy of intravesical MMC plus BCG therapy for NMIBC patients up to June 2016. Search terms were “‘mitomycin C’ or ‘MMC’” and “‘bacillus Calmette-Guerin’ or ‘BCG’” in combination with “‘non-muscle-invasive bladder cancer’ or ‘NMIBC’ or ‘superficial bladder cancer’ or ‘orthotopic bladder cancer’ or ‘bladder carcinoma *in situ*’”. The study language was restricted to English and Chinese. Reference lists of relevant studies were also checked.

### Inclusion and exclusion criteria

Studies applying intravesical MMC plus BCG therapy on NMIBC patients and providing detailed information were included in this systematic review, and data comparing the efficacy of combination therapy with MMC or BCG monotherapy was pooled in cumulative analysis. Accordingly, we excluded studies involving congress abstracts, conference proceedings, editorials, reviews, animal experiments and repeated publications. Two authors (T.D. and B.L.) independently assessed relevant records, evaluated the quality of included studies and extracted studies’ data. Discrepancies were resolved via open discussion.

### Study quality assessment and data extraction

GRADE approach was used to assess the LOE of all eligible studies^43^. Furthermore, the Cochrane Collaboration Risk of Bias Tool was applied to evaluate the quality of RCTs^44^. Data was attentively extracted including research methodology, participants’ information, tumor stage, surgical procedure, therapeutic regimens of MMC plus BCG (instillation schedule, dose and retaining time), course of treatment, and disease-related outcomes (recurrence, progression, disease-free survival, disease-free interval, cancer-specific survival, overall survival and severe side-effect). In comparative studies, HRs and 95% CIs were also extracted to predict the recurrence-free survival between combined MMC plus BCG and MMC or BCG alone.

### Statistics analysis

In the cumulative analysis, summarized unadjusted ORs and 95% CIs were calculated to assess the efficacy of combined MMC and BCG instillation compared with MMC or BCG alone. Available multivariable adjusted HRs were also pooled as references. Subgroup analyses were conducted according to type of combination regimen, study design, patient ethnicity, number of instillation, therapeutic course, and follow-up time. Statistical heterogeneity among included studies was tested through chi-square test^45^. If no heterogeneity existed with *p* value > 0.10, the fixed-effect model was used. Otherwise, the random-effect model was applied. A two-sided *p* value < 0.05 was considered significant for all results in cumulative analysis. Publication bias was assessed by inverted funnel plot and Egger’s test^46^. All statistical analyses were conducted by RevMan (version 5.3; Cochrane Collaboration, Oxford, UK) and STATA (version 13.0; StataCorp, College Station, Texas, USA) software.

## Electronic supplementary material

Dataset 1
