# Supplementary material for: Systematic Review and Cumulative Analysis of the Combination of Mitomycin C plus Bacillus Calmette-Guérin (BCG) for Non–Muscle-Invasive Bladder Cancer
Source: Sci Rep. 2017 Jun 9;7:3172. doi: 10.1038/s41598-017-03421-5 (PMC5466691; doi:10.1038/s41598-017-03421-5)

**Systematic Review and Cumulative Analysis of the Combination of Mitomycin C plus Bacillus Calmette-Guérin (BCG) for Non–Muscle-invasive Bladder Cancer**

Tuo Deng^1, #^, Bing Liu^2, #^, Xiaolu Duan^1, #^, Tao Zhang^1^, Chao Cai^1^, Guohua Zeng^1, *^

1. Department of Urology, Minimally Invasive Surgery Center, The First Affiliated Hospital of Guangzhou medical University. Guangzhou Institute of Urology. Guangdong Key Laboratory of Urology, Guangzhou, China.

2. The First Affiliated Hospital of Jinan University, Guangzhou, China.

#: These authors contribute equally to this work.

*Corresponding author: Guohua Zeng

Department of Urology, Minimally Invasive Surgery Center, the First Affiliated Hospital of Guangzhou Medical University. Guangzhou Institute of Urology. Guangdong Key Laboratory of Urology.

Address: Kangda Road 1#, Haizhu District, Guangzhou, Guangdong, China, 510230

Telephone: +86-020-34294145

Email: [gzgyzgh@vip.tom.com](mailto:gzgyzgh@vip.tom.com)

**Supplementary Table 1.**  Comparison of patients receiving combination therapy and BCG monotherapy

| *Reference* | *No. of cases* | | | *Mean/ median age (yr)* | | *Dose and retaining time* | | | *Instillation schedule* | | *Total instillation number and duration (mo)* | *Mean/ median follow-up time (mo)* | *During follow-up time* | | | | | | *5-year recurrence-free survival rate* | *5-year overall survival rate* | *No. severe side- effects* |
| --- | --- | --- | --- | --- | --- | --- | --- | --- | --- | --- | --- | --- | --- | --- | --- | --- | --- | --- | --- | --- | --- |
|  |  |  |  |  |  | *MMC* | *BCG* | |  |  |  |  | *No. recurrence* | *No. disease-free case* | *Mean/ median recurrence- free interval (mo)* | *No. progression* | *No. death from any causes* | *No. death from bladder cancer* |  |  |  |
| **A single dose of perioperative MMC prior to instillation with BCG** | | | | | | | | | | |  |  |  |  |  |  |  |  |  |  |  |
| Badalato et al, 2011 [14] | | MMC+BCG | 48 | | 69.6 | 40 mg; - | | - | | - | - | 33 | 21 | 27 | - | - | - | - | 56.3% | - | - |
|  |  | BCG | 212 | |  | - | | - | | - | - | 43.6 | 132 | 80 | - | - | - | - | 37.5% | - | - |
| Gülpinar et al, 2012 [10] | | MMC+BCG | 25 | | 58.2 | 40 mg/40 ml ; 60-120 min | | 5 × 10^8^ CFU/50 ml; 60-120 min | | MMC was administered within 6 hours of surgery. BCG was administered at least 15 days from surgery and once a week for 6 weeks | 7; 1.9 | 41.3 | 9 | 16 | 8 | 1 | - | - | - | - | 0 |
|  |  | BCG | 26 | | 58 | - | | 5 × 10^8^ CFU/50 ml; 60-120 min | | BCG was administered at least 15 days from surgery and once a week for 6 weeks | 6; 1.9 | 40.9 | 5 | 21 | 7 | 1 | - | - | - | - | 0 |
| Weiss et al, 2015 [29] | | MMC+BCG | 23 | | - | 40 mg; - | | - | | - | - | 54 | 10 | 10 | - | - | 3 | - | 48.4% | 87.5% | - |
|  |  | BCG | 97 | |  | - | | - | | - | - |  | 42 | 43 | - | - | 12 | - | 50.1% | 88% | - |
| **Sequential instillation with MMC and BCG** | | | | | |  | |  | |  |  |  |  |  |  |  |  |  |  |  |  |
| Di Stasi et al, 2006 [11] | | MMC+BCG | 107 | | 66 | 40 mg/100 ml; 60 min | | 10.2±9.0×10 ^8^ CFU/50 ml; 120 min | | One course of treatment per week for 9 weeks (one cycle consisted of two BCG infusions and one mitomycin infusion, three cycles in total). Patients who were disease-free for 3 months after treatment were scheduled to receive one infusion a month for 9 months: three cycles of mitomycin, mitomycin, and BCG | 18; 11.1 | 91 | 45 | 62 | 69 | 10 | 23 | 6 | - | - | 3 |
|  |  | BCG | 105 | | 67 | - | | 10.2±9.0×10^8^ CFU/50 ml; 120 min | | One course of intravesical treatment per week for 6 weeks. Patients who were disease-free for 3 months after treatment were scheduled to receive monthly infusion of BCG for 10 months | 16; 11.4 | 84 | 61 | 44 | 21 | 23 | 34 | 17 | - | - | 3 |
| Oosterlinck et al, 2011 [16] | | MMC+BCG | 41 | | 68 | 40 mg/50 ml; - | | 5 × 10^8^ CFU/50 ml; - | | Weekly MMC for 6 weeks followed by six weekly BCG instillations. Complete responders after induction received three weekly maintenance instillations (one instillation of MMC followed by two BCG instillations weekly) at 6, 12, 18, 24, 30 and 36 mo | 12; 2.8 | 56.4 | 23 | 25 | - | 3 | 7 | 0 | 51.4% | 82.7% | 5 |
|  |  | BCG | 42 | | 70 | - | | 5 × 10^8^ CFU/50 ml; - | | Six weekly BCG instillations followed by 3 weeks of rest and 3 weeks of BCG. Complete responders after induction received three weekly maintenance instillations (BCG) at 6, 12, 18, 24, 30 and 36 mo | 9; 2.1 |  | 26 | 22 | - | 14 | 11 | 6 | 43.6% | 77.8% | 7 |
| He et al, 2013 [17] | | MMC+BCG | 40 | | 61.2 | 40 mg/30 ml; 2 h | | 1 mg/30 ml; 2 h | | Daily intravesical MMC instillation for one week followed by six weekly low dose BCG instillations and 12 monthly BCG instillations. If no recurrence occurred, patients would receive three more BCG instillations for each 3 months | 28; 22.6 | 21.2 | 5 | - | - | - | - | 0 | - | - | 0 |
|  |  | BCG | 39 | |  | - | | 1 mg/30 ml; 2 h | | Six weekly low dose BCG instillations and 12 monthly BCG instillations. If no recurrence occurred, patients would receive three more BCG instillations for each 3 months | 21; 22.4 |  | 10 | - | - | - | - | 0 | - | - | 0 |
| Liu et al, 2007 [18] | | MMC+BCG | 59 | | 55 | 20 mg/20 ml; 2 h | | 150 mg/50 ml; 2 h | | Three intravesical MMC instillations at 6 h, one week and two weeks after operation. Then 6 weekly intravesical BCG instillations would start from the 3rd week, followed by three weekly BCG instillations at 3, 6, 12, 18, 24, 30 and 36 mo | 30; 36 | 35 | 9 | - | - | 3 | - | 0 | - | - | 0 |
|  |  | BCG | 51 | |  | - | | 150 mg/50 ml; 2 h | | Six weekly intravesical BCG instillations followed by three weekly BCG instillations at 3, 6, 12, 18, 24, 30 and 36 mo | 27; 36 |  | 17 | - | - | 4 | - | 0 | - | - | 0 |
| **Alternating instillation with MMC and BCG** | | | | | |  | |  | |  |  |  |  |  |  |  |  |  |  |  |  |
| Kaasinen et al, 2003 [24] | | MMC+BCG | 159 | | 71 | 40 mg/50 ml; - | | 120 mg/50 ml; - | | Six weekly intravesical instillations of MMC followed by alternating instillations of BCG and MMC monthly for one year | 18; 13.4 | 56.3 | 71 | 72 | 39.1 | 34 | - | 13 | 40.7% | - | 10 |
|  |  | BCG | 145 | | 70 | - | | 120 mg/50 ml; - | | Six weekly BCG instillations followed by monthly BCG instillations for one year | 18; 13.4 |  | 53 | 80 | - | 20 | - | 10 | 53.8% | - | 37 |
| Zhang et al, 2009 [25] and Sun et al, 2010 [26] | | MMC+BCG | 32 | | 62.5 | 40 mg/40 ml; 2 h | | 120 mg/40 ml; 2 h | | Alternating intravesical BCG and MMC instillation once a week for 8 weeks and then once a month for 10 months, followed by once 3 months for one year | 22; 24 | 28 | 2 | 30 | 26.9 | 0 | 0 | 0 | - | - | 0 |
|  |  | BCG | 22 | |  | - | | 120 mg/40 ml; 2 h | | Weekly intravesical BCG instillation for 8 weeks and then once a month for 10 months, followed by once 3 months for one year | 22; 24 |  | 5 | - | - | - | - | 0 | - | - | 3 |
| Bao et al, 2007 [27] | | MMC+BCG | 20 | | 70 | 20 mg/50 ml; 2 h | | 120 mg/50 ml; 2 h | | Alternating intravesical MMC and BCG instillation once a week for 6 weeks and then once 2 weeks for 12 weeks, followed by once a month for 20 months | 32; 24 | 24 | 0 | - | - | 0 | - | 0 | - | - | 0 |
|  |  | BCG | 18 | | 72 | - | | 120 mg/50 ml; 2 h | | Intravesical BCG instillation once a week for 6 weeks and then once 2 weeks for 12 weeks, followed by once a month for 20 months | 32; 24 |  | 3 | - | - | 1 | - | 1 | - | - | 0 |
| **Mixed instillation with MMC plus BCG** | | | | | | | | | |  |  |  |  |  |  |  |  |  |  |  |  |
| Solsona et al, 2015 [13] | | MMC+BCG | 211 | | 65 | 10 or 30 mg/50 ml; 1 h | 1.5-5 × 10^8^ CFU/50 ml; 1 h | | | Weekly intravesical BCG instillation for 6 weeks, with a dose of MMC the previous day, followed by three more instillations 2 wk apart | 18; 2.6 | 85.2 | 44 | - | - | 26 | 51 | 10 | - | - | 20 |
|  |  | BCG | 196 | | 66 | - | 1.5-5 × 10^8^  CFU/50 ml; 1 h | | | Weekly intravesical BCG instillation for 6 wk followed by three more instillations 2 wk apart | 9; 2.6 |  | 68 | - | - | 24 | 52 | 15 | - | - | 9 |

BCG = bacillus Calmette-Guerin; MMC = mitomycin C.

**Supplementary Table 2.** Subgroup analyses of tumor recurrence rates comparing combination therapy with BCG monotherapy

| *Subgroup* | | *Number of studies* | *Heterogeneity* | | *OR (95%CI)* |
| --- | --- | --- | --- | --- | --- |
|  |  |  | I^2^ | P |  |
| Study design | RCT | 7 | 67% | 0.005 | 0.70 (0.44, 1.12) |
|  | Retrospective comparative trial | 4 | 25% | 0.26 | **0.52 (0.32, 0.84)** |
| Combination regimen | Combination regimen 1 | 3 | 64% | 0.06 | 0.91 (0.38, 2.20) |
|  | Combination regimen 2 | 4 | 0% | 0.66 | **0.52 (0.35, 0.76)** |
|  | Combination regimen 3 | 3 | 69% | 0.04 | 0.48 (0.09, 2.51) |
|  | Combination regimen 4 | 1 | NA | | **0.50 (0.32, 0.77)** |
| Follow-up time | Short-term (≤ 2 yrs) | 2 | 0% | 0.41 | **0.33 (0.11, 0.96)** |
|  | Medium-term (2-5 yrs) | 7 | 63% | 0.01 | 0.77 (0.45, 1.31) |
|  | Long-term (≥ 5 yrs) | 2 | 0% | 0.88 | **0.51 (0.36, 0.71)** |
| Ethnicity | Europeans | 5 | 74% | 0.004 | 0.83 (0.48, 1.44) |
|  | Asians | 4 | 0% | 0.83 | **0.33 (0.17, 0.62)** |
|  | Mixed | 2 | 44% | 0.18 | 0.60 (0.36, 1.02) |
| Therapeutic course | ≤ 1 yr | 4 | 48% | 0.12 | **0.59 (0.43, 0.80)** |
|  | 1 - 2 yrs | 4 | 67% | 0.03 | 0.51 (0.16, 1.59) |
|  | ＞ 2 yrs | 1 | NA | | **0.36 (0.14, 0.90)** |
|  | Unknown | 2 | 44% | 0.18 | 0.60 (0.36, 1.02) |
| Instillation number | ≤ 12 | 2 | 49% | 0.16 | 1.13 (0.56, 2.29) |
|  | 12-24 | 4 | 78% | 0.003 | 0.64 (0.33, 1.23) |
|  | ≥ 24 | 3 | 0% | 0.72 | **0.34 (0.17, 0.69)** |
|  | Unknown | 2 | 44% | 0.18 | 0.60 (0.36, 1.02) |

RCT = randomized controlled trial; OR = odds ratio; CI = confidence interval.

Combination regimen 1: a single dose of perioperative MMC prior to BCG; Combination regimen 2: sequential instillation with MMC and BCG; Combination regimen 3: alternating instillation with MMC and BCG; Combination regimen 4: mixed instillation with MMC plus BCG.

The bold numbers mean the P-value is < 0.05.

**Supplementary Table 3. C**omparison of patients receiving combination therapy and MMC monotherapy

| *Reference* | | *No. of case* | | *Mean/ median age(yr)* | | | *Dose and retaining time* | | | | *Instillation schedule* | | *Total instillation number and duration (mo)* | | *Mean/ median follow-up time (mo)* | | *During follow-up time* | | | | | | | | | | | | *5-year recurrence-free survival* | | *No. severe side- effects* | |  | |
| --- | --- | --- | --- | --- | --- | --- | --- | --- | --- | --- | --- | --- | --- | --- | --- | --- | --- | --- | --- | --- | --- | --- | --- | --- | --- | --- | --- | --- | --- | --- | --- | --- | --- | --- |
|  |  |  |  |  |  |  | *MMC* | *BCG* | | |  |  |  |  |  |  | *No. recurrence* | | *No. disease-free case* | | *Mean/median disease/recurrence-free interval (mo)* | | *No. progression* | | *No. death from any causes* | | *No. death from bladder cancer* | |  |  |  |  |  | |
| **A single dose of perioperative MMC prior to instillation with BCG** | | | | | | | | | | |  | |  | |  | |  | |  | |  | |  | |  | |  | |  | |  | |  | |
| Ye et al, 2003 [15] | MMC+BCG | | 50 | | 57 | | 40 mg/30 ml; 2 h | | | 1 mg/30 ml; 2 h | | An immediate intravesical MMC instillation after operation followed by six weekly low dose BCG instillations and 12 monthly BCG instillations. If no recurrence occurred, patients would receive three more BCG instillations for each 3 months | | 22; 22.6 | | 32 | | 3 | | - | | - | | 0 | | - | | 0 | | - | | 0 | |  |
|  | MMC | | 33 | |  |  | 30 mg/30 ml; 2 h | | | - | | Daily intravesical MMC instillation from 7th to 14th day after operation | | 7; 0.2 | |  |  | 7 | | - | | - | | 1 | | - | | 0 | | - | | 1 | |  |
| **Sequential instillation with MMC and BCG** | | | | | | | | |  | |  | |  | |  | |  | |  | |  | |  | |  | |  | |  | |  | |  | |
| Ma et al, 2001 [19] | MMC+BCG | | 29 | | 52 | | 30 mg/50 ml; 2 h | | | 120 mg/50 ml; 2 h | | Weekly intravesical MMC instillation for 4 weeks followed by intravesical BCG instillations weekly for 6 weeks and monthly for 2 years | | 34; 26.3 | | 37.9 | | 3 | | - | | - | | - | | - | | - | | - | | 0 | |  |
|  | MMC | | 29 | |  |  | 30 mg/50 ml; 2 h | | | - | | Intravesical MMC instillation weekly for 6 weeks and monthly for 2 years | | 30; 25.4 | |  |  | 11 | | - | | - | | - | | - | | - | | - | | 2 | |  |
| Witjes et al, 1998 [31] | MMC+BCG | | 90 | | - | | 40 mg/50 ml; - | | | 5 x 10E8 CFU/50 ml; - | | 4 weekly instillations with 40 mg. mitomycin C followed by 6 weekly instillations with BCG | | 10; 2.3 | | 32 | | 35 | | 47 | | 43.7 | | 5 | | 21 | | 5 | | 52.2% | | 16 | |  |
|  | MMC | | 92 | |  |  | 40 mg/50 ml; - | | | - | | 4 weekly instillations with 40 mg. mitomycin C followed by 10 weekly instillations with mitomycin C | | 14; 3.3 | |  |  | 42 | | 32 | | - | | 4 | | 14 | | 8 | | 34.8% | | 8 | |  |
| **Alternating instillation with MMC and BCG** | | | | | | | | |  | |  | |  | |  | |  | |  | |  | |  | |  | |  | |  | |  | |  | |
| Rintala et al, 1995 [33] and Järvinen et al, 2012 [12] | MMC+BCG | | 28 | | 66 | | adjusted according to the bladder capacity; - | | | 6 × 10^8^ CFU/50 ml; - | | An induction period (a first MMC instillation perioperatively or at least within 2 days of TUR, followed by four additional weekly instillations of MMC) followed by monthly instillations of alternating MMC and BCG instillations during the first year and every third month during the second year | | 21; 24 | | 33 | | 6 | | 14 | | - | | 2 | | 0 | | 0 | | - | | 0 | |  |
|  |  |  |  |  |  |  |  |  |  |  |  |  |  |  |  | 86.4 | | 19 | | - | | - | | 8 | | 20 | | 8 | | 34% | | - | |  |
|  | MMC | | 40 | | 68 | | adjusted according to the bladder capacity; - | | | - | | An induction period (a first MMC instillation perioperatively or at least within 2 days of TUR, followed by four additional weekly instillations of MMC) followed by monthly instillations of MMC alone during the first year and every third month during the second year | | 21; 24 | | 33 | | 17 | | 11 | | - | | 4 | | 2 | | 0 | | - | | 2 | |  |
|  |  |  |  |  |  |  |  |  |  |  |  |  |  |  |  | 86.4 | | 35 | | - | | - | | 14 | | 30 | | 12 | | 20% | | - | |  |
| Zhang et al, 2009 [25] and Sun et al, 2010 [26] | MMC+BCG | | 32 | | 62.5 | | 40 mg/40 ml; 2 h | | | 120 mg/40 ml; 2 h | | Alternating intravesical BCG and MMC instillation once a week for 8 weeks and then once a month for 10 months, followed by once 3 months for another year | | 22; 24 | | 28 | | 2 | | 30 | | 26.9 | | 0 | | 0 | | 0 | | - | | 0 | |  |
|  | MMC | | 24 | |  |  | 40 mg/40 ml; 2 h | | | - | | Weekly intravesical MMC instillation for 8 weeks and then once a month for 10 months, followed by once 3 months for another year | | 22; 24 | |  |  | 7 | | - | | - | | - | | - | | 0 | | - | | 0 | |  |
| Rintala et al, 1996 [34] | MMC+BCG | | 95 | | 68 | | adjusted according to the bladder capacity; - | | | 6 x 10E8 CFU/50 ml; - | | An induction period (a first MMC instillation perioperatively or at least within 2 days of TUR, followed by four additional weekly instillations of MMC) followed by monthly instillations of alternating MMC and BCG instillations during the first year and every third month during the second year | | 21; 24 | | 34 | | 57 | | 38 | | 6.9 | | 3 | | 2 | | 0 | | - | | 6 | |  |
|  | MMC | | 93 | | 69 | | adjusted according to the bladder capacity; - | | | - | | An induction period (a first MMC instillation perioperatively or at least within 2 days of TUR, followed by four additional weekly instillations of MMC) followed by monthly instillations of MMC alone during the first year and every third month during the second year | | 21; 24 | |  |  | 58 | | 35 | | 12.4 | | 3 | | 4 | | 0 | | - | | 6 | |  |
| **Mixed instillation with MMC plus BCG** | | | | | | | | | | |  | |  | |  | |  | |  | |  | |  | |  | |  | |  | |  | |  | |
| Fang et al, 2002 [28] | MMC+BCG | | 21 | 67.5 | | MMC 20 mg + BCG 2 mg/40 ml; 2 h | | | | | | Weekly intravesical mixed MMC plus BCG instillation for 6 weeks followed by once a month for 2 years | | 30; 25.4 | | 23.4 | | 1 | | - | | - | | - | | - | | 0 | | - | | 0 | |  |
|  | MMC | | 21 | 66.5 | | 20 mg/40 ml; 2 h | | | | - | | Weekly intravesical MMC instillation for 6 weeks followed by once a month for 2 years | | 30; 25.4 | | 19.3 | | 7 | | - | | - | | - | | - | | 0 | | - | | 0 | | |

BCG = bacillus Calmette-Guerin; MMC = mitomycin C.

**Supplementary Figure legends:**

Supplementary Figure 1: Forest plot of disease-free survival rate comparing combination therapy with BCG monotherapy

Supplementary Figure 2: Forest plot of cancer-specific mortality comparing combination therapy with BCG monotherapy

Supplementary Figure 3: Forest plot of toxicities comparing combination therapy with BCG monotherapy

Supplementary Figure 4: Forest plot of tumor progression rate comparing combination therapy with MMC monotherapy

Supplementary Figure 5: Forest plot of toxicities comparing combination therapy with MMC monotherapy

**Supplementary Figure 1**. Forest plot of disease-free survival rate comparing combination therapy with BCG monotherapy

**
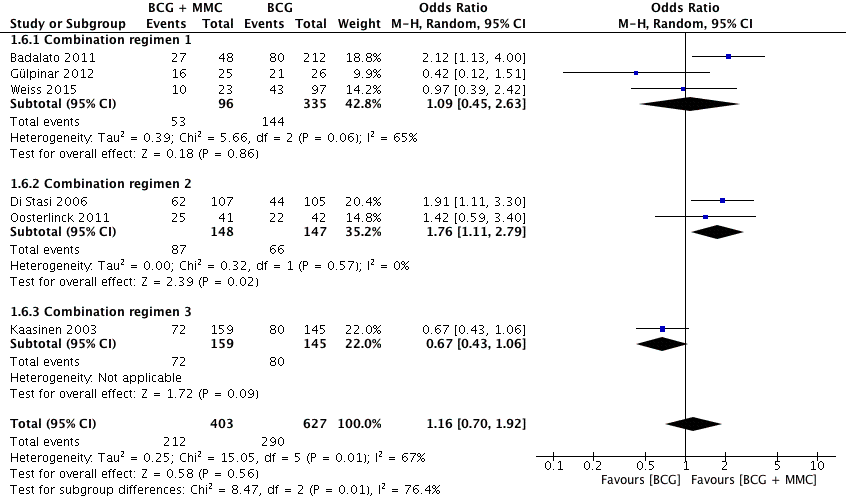
**

**Supplementary Figure 2**. Forest plot of cancer-specific mortality comparing combination therapy with BCG monotherapy


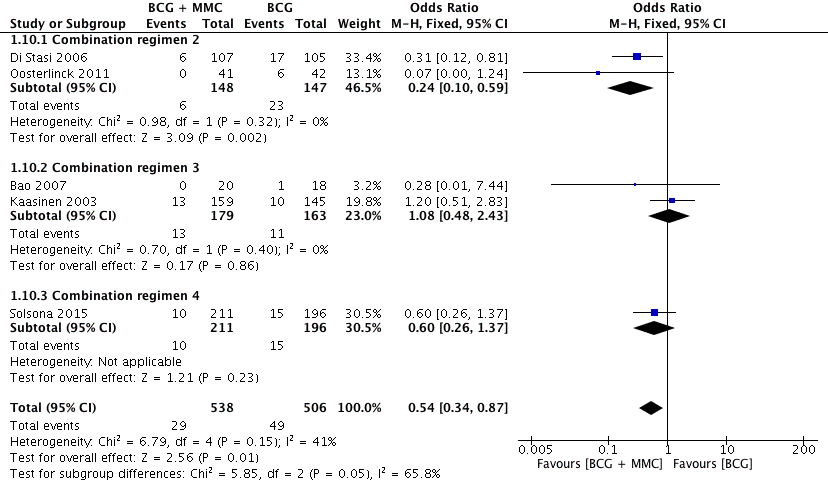


**Supplementary Figure 3.** Forest plot of toxicities comparing combination therapy with BCG monotherapy


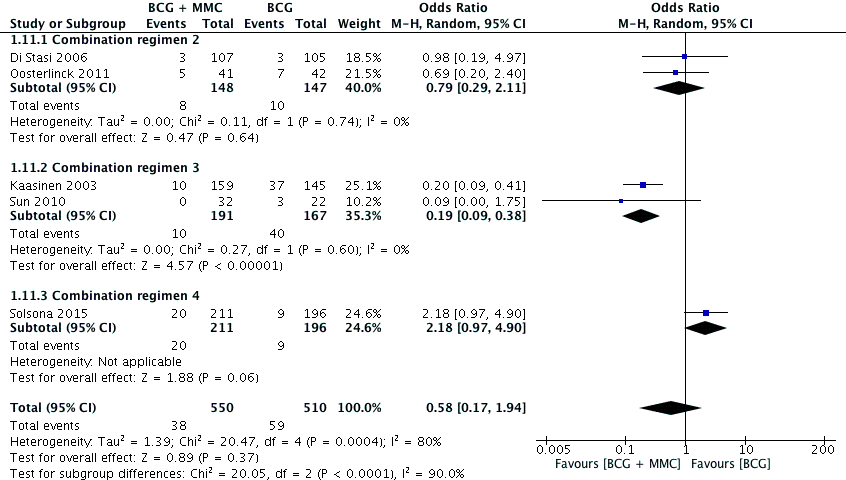


**Supplementary Figure 4.** Forest plot of tumor progression rate comparing combination therapy with MMC monotherapy


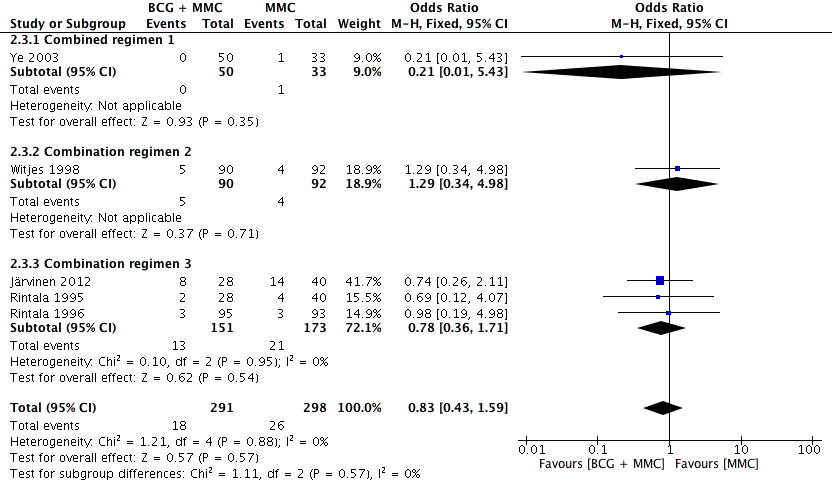


**Supplementary Figure 5.** Forest plot of toxicities comparing combination therapy with MMC monotherapy


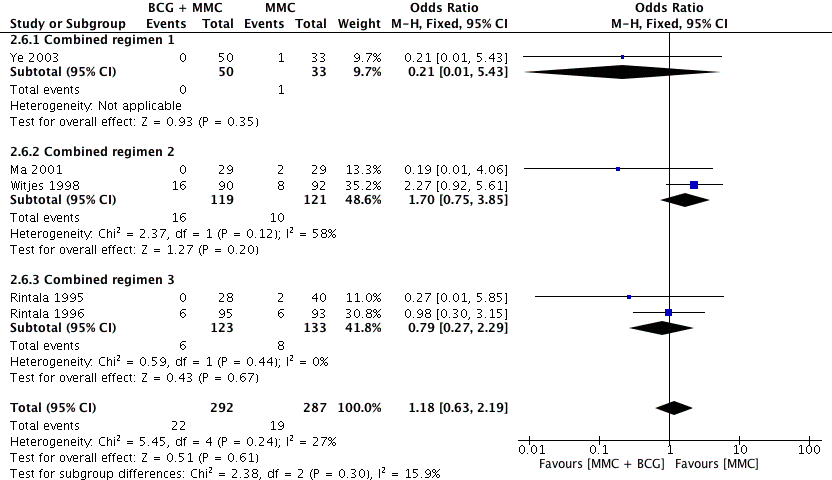

Supplement: Supplementary file 1 — Dataset 1 [file 41598_2017_3421_MOESM1_ESM.doc]
